# Expansion of aerobic methanotrophy to the phylum of Actinomycetota and its environmental implications

**DOI:** 10.1128/aem.01334-25

**Published:** 2025-09-08

**Authors:** Paul L.E. Bodelier

**Affiliations:** 1 Department of Microbial Ecology, Netherlands Institute of Ecology (NIOO-KNAW) 3886 , Wageningen, the Netherlands; Michigan State University, East Lansing, Michigan, USA

**Keywords:** methanotrophy, Mycobacteria, methane, Actinomycetota

## Abstract

For over a century, taxonomically validated pure cultures of aerobic methanotrophs belonged to Pseudomonadota, or since 2007, Verrucomicrobiota. A recent article published in *Applied and Environmental Microbiology* by H. Kambara, T. Kawamoto, S. Matsushita, T. Kindaichi, et al. (91:e00796-25, 2025, https://doi.org/10.1128/aem.00796-25) expands the phylogenetic radiation of aerobic methanotrophs to the Actinomycetota with the description of *Ca*. Mycobacterium methanica MM1. This isolate confirms and strengthens the position of the genus *Mycobacterium* as methanotrophs displaying a wide pH and ammonia tolerance and expanding the habitat range for methane capture.

## COMMENTARY

The climate crisis is one of the most imminent threats to humanity and all life on our planet. Methane is not only the second most potent greenhouse gas (GHG) and is responsible for approximately 31% of GHG-induced global warming since the start of the industrial revolution (1) but is also the most rapid increasing GHG in the atmosphere (2). The latter is likely caused by increased microbiological production of methane from, e.g., wetlands (2). This fact stresses the importance of methanotrophic microbes that can degrade methane with and without oxygen (3, 4) and thereby fulfill a crucial role in our global climate.

However, the consumption of methane is a trait that is only described for microbial strains belonging to a limited number of phyla. The contribution of H. Kambara (5) (“First isolation of a methanotrophic *Mycobacterium* reveals ammonia- and pH-tolerant methane”) expands the radiation of isolated aerobic methanotrophs into the phylum of the Actinomycetota, with the description of a *Mycobacterium* species capable of growth on methane as the sole carbon and energy source.

The first report of aerobic bacteria growing on methane in 1906 (6) taxonomically described isolates in the phylum of the Pseudomonadota (Alphaproteobacteria and Gammaproteobacteria). A century later, in 2007, two research groups described in back-to-back publications the first isolates in the phylum of Verrucomicrobiota growing on methane aerobically under extreme temperature and pH conditions of geothermal habitats (7, 8). These microbes, among several other isolates, are now classified as members of the family of *Methylacidiphylaceae* (9).

It is now, 120 years after the description of “Bacillus methanicus,” that methanotrophy expands “officially” into the phylum of Actinomycetota with the isolation of *Ca*. Mycobacterium methanica. Such a discovery is surprising considering that mycobacterial strains were first reported to grow on methane in 1949 (10). Unfortunately, none of these strains have been preserved nor thoroughly tested for purity and physiology, leaving a void of knowledge, filled partially only in 2022 with the description of *Ca*. Mycobacterium methanotrophicum (hereafter M. methanotrophicum) (11). This highly enriched culture obtained from a cave biofilm harbors an extremophilic *Mycobacterium*, growing extremely slowly at pH 1.5 (doubling times approx. 60 days) in the presence of a fungus, which to date has impeded the recovery of this strain in pure culture as well as the investigation and verification of its physiology. *Ca*. Mycobacterium methanica strain MM1 (hereafter M. methanica) (5) promises to be a game changer, being pure and not too difficult to grow. This will allow the production of sufficient biomass for extensive morphological, genetic, as well as physiological testing, facilitating a taxonomic description and deposition at culture collections. The latter is necessary, not only to make the strain available to the academic community but also to go beyond genomic/proteomic predictions of the potential of the organisms and perform actual experiments for hypothesis testing. This is highly necessary because the new isolate (5) was recovered as a pure culture and tested for growth, pH and temperature range, and ammonium tolerance. However, basic morphological, genetic, and physiological features need further exploration.

Despite these gaps in knowledge, it is tempting to speculate on a few typical similarities between M. methanica and M. methanotrophicum that are striking. Both strains only carry the cytoplasmic, soluble methane monooxygenase (sMMO). Most described methanotrophs have only the membrane-bound particulate form of the enzyme (pMMO) or both sMMO and pMMO (12). *Methylocella* and *Methyloferula*, both sMMO-only possessing genera, were isolated from acidic environments, which may be a parallel to M. methanica (optimum pH for growth = 4), while M. methanotrophicum grows at pH 1.5.

Both strains putatively use a different enzyme for the conversion of methanol to formaldehyde than all other methanotrophs. Instead of the MxaF or XoxF type methanol dehydrogenases, both strains possess an alcohol dehydrogenase (Adh) proposed to carry out the conversion of methanol. The Adh in M. methanotrophicum has similarities with the one found in *Mycobacterium smegmatis* (11), which has been demonstrated to carry out methanol conversion. Next to this, both strains assimilate carbon via the ribulose monophosphate pathway, grow slowly, and have no internal membrane structures, which are typical for proteobacterial methanotrophs. Both Mycobacterial methanotrophs are very tolerant to extreme conditions, low and high pH, high ammonium (M. methanica), low pH and high sulfide (M. methanotrophicum), and both were growing and isolated from biofilms (from cave walls and sponges in a sewage treatment reactor).

It is tempting to speculate whether these microbes have been subjected to similar evolutionary pressures. In both studies with M. methanica and M. methanotrophicum, the authors have assessed the global occurrence of these microbes by searching databases for matches using the 16S rRNA gene sequence. Of course, this does not mean that microbes associated with the matched sequences are also able to consume methane, but some interesting parallels can be observed. Along with volcanic lava beds and soil, M. methanotrophicum was found in biofilms of corroded sewer systems (13). M. methanica is predominantly detected in freshwater habitats, in biofilms in drinking water production systems, host-associated (guts of fish and insects), and in water in pitcher plants.

Hence, the growth in biofilms may be a common denominator as well as the association with freshwater, assuming that M. methanotrophicum ended up in the biofilm on the cave walls in Romania by water that at some point entered the cave. The biofilm in “sulfur cave” in Romania (11, 14), as well as the biofilms in the “sponge-reactor” studies of H. Kambara (5, 15), harbor multiple *Mycobacterium* species, none of which have ended up in enrichments or isolations using methane. Apparently, they do not have the full genetic machinery to respire and assimilate methane carbon. It is well known that members of the family *Mycobacteriaceae* can degrade a broad range of alkanes, among which are gaseous ones such as ethane, propane, and butane (16), and also carry subunits of the sMMO gene (17). Nevertheless, as yet, methanotrophic Mycobacteria in culture or in metagenome-assembled genomes (MAGs) are very rare despite the fact that monooxygenases are subject to frequent lateral gene transfer (17). However, the ability to respire and assimilate methane requires the acquisition of a complex set of genes, which may only happen under very specific conditions, such as enclosure in biofilm matrices, where close proximity of species for extended time periods facilitates gene transfer.

To continue these lines of speculation, it is tempting to reflect on the environmental implications of the findings of H. Kambara (5). Mycobacteria and other members of the Actinomycetota, like Streptomycetes, are metabolically extremely versatile. Their potential participation in environmental methane degradation would change our perspective on controls of this process drastically, as they are ubiquitous in many environments and do not depend on methane as a sole carbon and energy source. Hence, the range of environmental factors regulating methane consumption, which traditionally has been focused on the presence of proteobacterial methanotrophs (3, 4), will have to be expanded, as will the methods of assessment. Primer-based studies using traditional sMMO primers do not pick up the divergent Mycobacterial gene sequences.

As yet, there are indications of methane assimilation in environmental settings, of which the earlier study of H. Kambara (15) is the most convincing. Next to this, highly negative ^13^C-isotopic signatures of Mycobacterial membrane mycoserosic acids point toward incorporation of methane from a biogenic origin in geothermal gas-seep soils (18). Similarly, many isotope (^13^C-CH_4_) labeling studies have detected Mycobacteria in the heavy DNA-labeled fraction (19, 20), which can either indicate assimilation of methane carbon directly or incorporation of ^13^C-labeled carbon by cross feeding. The latter is mostly assumed, but given the existence of M. methanica and M. methanotrophicum, the possibility of “real” methanotrophy by Mycobacteria needs to be taken into consideration in future studies but also in many studies in retrospect.

A very interesting question from an environmental perspective is whether Mycobacteria can also be involved in atmospheric methane consumption. Mycobacteria have been demonstrated to be able to utilize atmospheric hydrogen and carbon monoxide (21), as can many other microbial phyla (22). Methanotrophs have recently been demonstrated to exhibit a mixtrophic lifestyle, utilizing and growing on a combination of atmospheric hydrogen, carbon monoxide, and methane (23). However, the tested species that display high-affinity methane oxidation contain the membrane-bound particulate form (pMMO), which is generally thought to have higher affinity, whereas the soluble form (sMMO) operates at higher methane concentrations, which is the form in Mycobacteria detected so far. However, the study of Schmider et al. (23) demonstrated that it can be a matter of not using the right methods and conditions to actually assess whether a strain displays high-affinity methane oxidation kinetics. Hence, for M. methanica, it remains to be tested whether it can utilize atmospheric methane, and this is now feasible with the availability of a pure culture.

The alternative would be Mycobacteria possessing pMMO, which to date have not been found. That, however, is no evidence that they do not exist, as the use of metagenomic screening studies has taught us. MAGs retrieved from dry land soils in Australia (22) contained *pmoA* sequences belonging to the uncultured USCγ cluster associated with high-affinity methane oxidation. These MAGs were phylogenetically assigned to the Gemmatimonadota, while in high Arctic soils incubated with low concentrations of methane, sMMO genes were retrieved in MAGs belonging to the phylum of Chloroflexota (19). These findings not only indicate that methanotrophy may be present in more phyla but also suggest potential involvement of sMMO-containing microbes in atmospheric methane consumption.

However, all these findings based on metagenomic evidence need to be verified, for which the golden standard is still a pure culture, which is exactly why the study of H. Kambara (5) is so special. Only a pure or highly enriched culture can give an answer to the most important question: Is a microbe associated with a MAG really capable of methanotrophy? The availability of a pure culture can also assist in designing more directed methods for detection in the environment, which is a necessary next step in assessing the contribution of Mycobacteria, or more generally the Actinomycetota, to global methane consumption. There are still many *pmoA*-related sequence types, which have no “parent” organism yet assigned to them, exemplified by USCγ cluster sequences dominating natural grasslands (24), caves (25), and arctic upland soils (26), and these may find their “home” in the Actinomycetota.

Undoubtedly, the methanotrophic “terra incognita” disclosed by the new isolate will spark many new studies that will lead to a more comprehensive understanding of the ecophysiology of methanotrophs and the regulation of environmental methane processing.
